# Expression Profiling and Functional Implications of a Set of Zinc Finger Proteins, ZNF423, ZNF470, ZNF521, and ZNF780B, in Primary Osteoarthritic Articular Chondrocytes

**DOI:** 10.1155/2014/318793

**Published:** 2014-05-27

**Authors:** Maria Mesuraca, Olimpio Galasso, Leonardo Guido, Emanuela Chiarella, Stefania Scicchitano, Renaud Vatrinet, Giovanni Morrone, Heather M. Bond, Giorgio Gasparini

**Affiliations:** ^1^Laboratory of Molecular Haematopoiesis and Stem Cell Biology, Department of Experimental and Clinical Medicine, University of Catanzaro Magna Græcia, University Campus “Salvatore Venuta”, Germaneto, 88100 Catanzaro, Italy; ^2^Orthopedic and Trauma Surgery, Department of Medical and Surgical Sciences, University of Catanzaro Magna Græcia, University Campus “Salvatore Venuta”, Germaneto, 88100 Catanzaro, Italy

## Abstract

Articular chondrocytes are responsible for the maintenance of healthy articulations; indeed, dysregulation of their functions, including the production of matrix proteins and matrix-remodeling proteases, may result in fraying of the tissue and development of osteoarthritis (OA). To explore transcriptional mechanisms that contribute to the regulation of chondrocyte homeostasis and may be implicated in OA development, we compared the gene expression profile of a set of zinc finger proteins potentially linked to the control of chondrocyte differentiation and/or functions (ZNF423, ZNF470, ZNF521, and ZNF780B) in chondrocytes from patients affected by OA and from subjects not affected by OA. This analysis highlighted a significantly lower expression of the transcript encoding ZNF423 in chondrocytes from OA, particularly in elderly patients. Interestingly, this decrease was mirrored by the similarly reduced expression of PPAR*γ*, a known target of ZNF423 with anti-inflammatory and chondroprotective properties. The ZNF521 mRNA instead was abundant in all primary chondrocytes studied; the RNAi-mediated silencing of this gene significantly altered the COL2A/COL1 expression ratio, associated with the maintenance of the differentiated phenotype, in chondrocytes cultivated in alginate beads. These results suggest a role for ZNF423 and ZNF521 in the regulation of chondrocyte homeostasis and warrant further investigations to elucidate their mechanism of action.

## 1. Introduction

Chondrocytes are virtually the only cellular component of the cartilage and have a role in maintaining the equilibrium between anabolic production of collagens type II and aggrecans which make up the extracellular matrix and catabolic enzymes, including aggrecanases and matrix metalloproteinases (MMPs), important for cartilage turnover [1]. In osteoarthritis (OA), a relative increase in the production of these enzymes can result in aberrant cartilage destruction. Autologous chondrocyte implantation (ACI) of ex vivo expanded chondrocytes retaining ability to repopulate focal lesions of articular cartilage [2] is an important therapeutic aim in orthopaedics. When cultured in supporting 3D scaffold substitute structures, chondrocytes maintain the production of collagen type II, aggrecans, and MMP13, whereas in monolayer cultures they display a progressive loss of these characteristic proteins and eventually gain a fibroblast-like phenotype and switch to collagen type I expression instead of type II, that reflects a dedifferentiated status. However, if chondrocytes from monolayer cultures are shifted to 3D scaffolds, they can reexpress chondrocyte-specific proteins and regain some degree of differentiation [3, 4]. In an effort to gain a better understanding of the molecular mechanisms that govern the phenotypes observed in OA and in the different culture conditions described above, that are still only partially known, we have investigated the expression of a group of zinc finger proteins potentially implicated in the physiological regulation of the homeostasis of articular chondrocytes, namely, ZNF423, ZNF470, ZNF521, and ZNF780B.

ZNF521/EHZF is a large multifunctional protein with 30 zinc fingers, identified in our laboratory for its selective abundance in immature hematopoietic progenitors compared to mature leukocytes [5, 6]. ZNF521 shows features of a transcriptional corepressor and has been found to modulate the transcriptional induction of erythroid and B-lymphoid differentiation by GATA1 and EBF1 [7, 8]. In addition to the hematopoietic system, ZNF521 has recently been demonstrated to drive the generation of neuroectodermal precursors from embryonic stem cells [9] and to contribute to the growth, clonogenicity, and tumorigenicity of medulloblastoma cells [10]. Importantly, the murine orthologue of ZNF521, termed Zfp521, has been identified as a central cell fate regulator in mesenchymal stem cells where, in a complex interplay with Ebf1 and Zfp423, it represses their adipogenic potential and promotes osteoblastic commitment [11, 12]. In osteoblasts, Zfp521 has been shown to be associated with Runx2 and antagonizes its transcriptional activity [13], thereby delaying their early differentiation steps and promoting later stages of osteoblast maturation. This activity is dependent on the interaction of Zfp521 with HDAC3 [14]. Relevantly, Zfp521 has also been shown to be an important downstream effector of PTHrP in growth plate chondrocytes in which it regulates proliferation and differentiation [15]. Zfp521 knock-out mice generated using the Cre recombinase driven by the collagen II promoter showed decreased growth plate chondrocyte proliferation, early hypertrophic transition, and reduced tibial growth plate thickness with less extracellular matrix. ZNF423/OAZ is the paralogue of ZNF521, with which it shares a high degree of homology (>66%), the structural architecture (including the presence of 30 zinc finger motifs), and some overlapping, but not necessarily identical, functions. This factor was originally identified as an inhibitor Olf1/Ebf1 in the olfactory epithelium [16–18]. It was subsequently found to be an important mediator in the signaling pathway of the bone morphogenetic proteins (BMP) 2 and 4 [19], as well as of retinoic acid [20] and of Notch [21]. ZNF423 is required for cerebellar development [22–24] and implicated in the development of B-lymphoid leukemias [25, 26]. Importantly, Zfp423 has recently risen to prominence as a master factor of the adipogenic commitment of mesenchymal stem cells through the activation of PPAR*γ* [27]. This effect is abrogated by WISP2 that binds to Zfp423 and sequesters it in the cytosol [28] and by Zfp52 which inhibits the Ebf1-mediated induction of Zfp423 expression in mesenchymal stem cells [11].

Two additional zinc finger proteins have been included in this study that are less well characterized but have both been linked to cartilage physiology. ZNF780B is an as yet uncharacterized zinc finger protein sharing 74.2% identity with mouse Zfp60, a zinc finger transcription factor with a KRAB domain and 19 zinc finger motifs. Zfp60 was originally identified in muscle differentiation [29]; its expression in cultured prehypertrophic chondrocytes coincides with the expression of regulators of chondrocyte maturation such as Indian Hedgehog (Ihh) and the PTHrP receptor, and its overexpression inhibits cartilage differentiation in the ATDC5 chondrogenic cell line [30]. ZNF470 is a protein with 17 zinc fingers as well as KRAB-A and KRAB-B motifs, whose mRNA was originally isolated from mesenchymal progenitors induced to differentiate towards chondrocytes in vitro. The ZNF470 transcript was found transiently expressed during chondrogenesis, with a peak of maximal abundance that coincided with the beginning of the increase in COL2A1 expression, and was strongly upregulated during the dedifferentiation of bovine articular chondrocytes in response to retinoic acid treatment [31].

Based on the evidence summarized above, these four zinc finger proteins can be considered likely candidate regulators of the homeostasis of differentiated articular chondrocytes that is compromised in OA. Therefore, we sought to determine whether the levels of their expression were altered in OA chondrocytes or in culture conditions that promote the emergence of a dedifferentiated phenotype that is reminiscent to some extent to that of osteoarthritic chondrocytes [32].

## 2. Materials and Methods

### 2.1. Cell Lines

The cell human chondrocyte cell lines T/C-28a4 and the C-28 derived from T/C-28a4 [33, 34] were cultured in adhesion in DMEM high glucose supplemented with 10% fetal bovine serum (FBS). The human hematopoietic cell lines, THP1 (acute monocytic leukemia), K562 (erythroleukemia), and IM9 (B cell lymphoma), were cultured in suspension in RPM1, 10% FBS; the medulloblastoma cell line DAOY was cultured in DMEM, 10% FBS. All media were supplemented with 100 U/mL penicillin and 50 *μ*g/mL streptomycin; the cells were maintained in 5% CO_2_ at 37°C. Tissue culture reagents were from Life Technologies (Italy).

### 2.2. Primary Articular Chondrocytes-Cartilage Specimens

The study protocol was approved by the local ethics committee and the research was carried out in compliance with the Declaration of Helsinki. Informed written consent was obtained from all individuals. Cartilage samples were obtained from a total of 25 osteoarthritic patients with an age range of 45–78 years having BMI values from 24.69 to 41.02. All patients were subjected to total joint replacement and samples of articular cartilage were obtained as discarded surgical material. Specifically, cartilage was taken from the femoral head in patients with hip OA and from femoral condyles and the tibial plateau in patients with knee OA [35]. Diagnosis of OA was based on established clinical and radiological criteria [36, 37]. A radiological classification of each osteoarthritic joint was carried out according to the Ahlbäck scale [38]. In addition, cartilage was harvested from 4 patients (3 underwent hallux valgus and 1 pes cavus deformity correction, resp.); these samples served as a control group (Table 1).

After the cartilage was removed from the joint, the tissue was extensively washed with isotonic saline solution to remove residual blood or synovial fluid. Specimens were excised from the superficial and deep layers of the articular cartilage avoiding the calcified layer and the subchondral bone. Cartilage fragments were cut finely, washed in phosphate buffered saline (PBS), and digested with 0.2% collagenase type 2 (Worthington) in DMEM high glucose for 16 h at 37°C. Debris was removed by filtration through 40 *μ*m cell strainers (BD Biosciences). The cell number and viability were assessed by 0.2% trypan blue and cells (3,000 cell/cm^2^) were allowed to adhere to collagen coated (dil 1 : 45 of 3 mg/mL Sigma) tissue culture dishes in DMEM high glucose, 10% FBS. Cells were passaged with 0.25% trypsin-EDTA at weekly intervals until 80% confluency on tissue culture dishes in DMEM 10% FBS for up to 5 passages.

### 2.3. RNA Extraction, cDNA Synthesis, and Q-RT-PCR

Total RNA was prepared with Trizol (Life Technologies) treated with DNase I (RNase free, Promega) 1 U/1 *μ*g RNA. The purity and amount of RNA were determined by spectrophotometric measurement (OD 260/280 ratio). cDNA was synthesized from 1 *μ*g RNA using SuperScript III reverse transcriptase at 42°C and 2.5 *μ*M random hexamers (Life Technologies). Q-PCR reactions were carried out with the iQ-SYBR green Supermix (Bio-Rad) in duplicate according to the manufacturer's instructions and analyzed using the iQ5 multicolor detection system (Bio-Rad). One cycle of 3 min at 95°C for activation was followed by 45 cycles of 10 seconds at 95°C, 10 seconds at 60°C, and 20 seconds at 72°C and then a melting curve. The specificity of amplification was confirmed by the dissociation curves of the amplicons and size of the amplified product analyzed on a 2% agarose gels. Relative gene expression was determined using the comparative threshold cycles Ct method, normalizing for endogenous GAPDH, and expression ratio was calculated as 2^−ddCt^. Results are then calculated relative to values from the T/C28a4 cell line. Standard deviations are calculated from the mean values obtained from the different tissue samples. Normalized expression values are reported in relative units RU and are used for comparative analysis among the samples.

Primers for chondrocyte markers, COL1, COL2A1, and aggrecan were as published [4] as well as those for MMP13 [39]. Primers for GAPDH and ZNF521 were as described [8]. Primers for the other zinc finger proteins were designed using the primer design program from NCBI with mpt 60–62°C spanning exon-intron junctions. Primers were for ZNF423 (fwd-ggaaaggcacccagacatcg, rev-cggggagtcgaacatctggt), for ZNF780B (fwd-ctagctgggggagaagcccga, rev-atcaggctgcaggcactccca), for ZNF470 (fwd-tgtgactgtccggtgcgtgg, rev-tgtgactgtccggtgcgtgg), and for PPAR*γ* (fwd-ctgtcggtttcagaagtgcct, rev-cccaaacctgatggcattgtgagaca).

### 2.4. Encapsulation of Chondrocytes in Alginate Beads

Chondrocytes grown in suspension in nonadherent conditions as spheres in Petri dishes for 24–48 h 1 × 10^6^/mL cells were encapsulated in 1 : 1 volume alginate solution (1.25% alginate acid (Sigma-Aldrich) 20 mM Hepes 150 mM NaCl pH 7.4) and polymerized by dropping, using a 21-gauge needle, into 102 mM CaCl_2_, 10 mM Hepes, pH 7.4. Beads were washed on 40 *μ*m strainers with 0.9% saline solution and cultured in DMEM 10% FBS in Petri dishes with weekly medium changes for 3 weeks [40]. Cells were recovered by treating beads with 55 mM EDTA in 10 mM Hepes pH7.4 and then RNA was prepared by Trizol reagent.

### 2.5. Lentiviral Transduction of shRNA

Lentiviral transduction was performed with chondrocytes encapsulated in alginate beads using lentiviral shRNA vectors expressing EGFP as a reporter marker, as described [8, 10, 41]. Viruses were prepared by cotransfecting 293T cells with plasmids coding for the viral components pCMV-VSVG and pCMV-deltaR8-91. After 48 h, viral supernatants were filtered (0.45 *μ*m) and added together with 4 *μ*g/mL polybrene to the chondrocyte cultures. The effectiveness of the transduction was monitored by visualization of the chondrocyte spheres for green fluorescent protein expression (FLOID microscope Life Technologies).

### 2.6. Immunofluorescence

Immunofluorescence  was performed on cells washed in PBS, fixed in 50% methanol and 50% acetone, dried, and washed in PBS followed by blocking in 10% FBS in PBS. Primary antibodies for COL2A1 sc2887 and ZNF521 (EHZF) sc84808 were from Santa Cruz BD Biosciences and were used at 1 : 200 for 16 hours and detected with anti-rabbit Alexa Fluor 498 (Life Technologies) at 1 : 200 binding for 2 hours. Cells were stained with DAPI for nuclear DNA staining. Images were acquired using the FLOID microscope from Life Technologies.

### 2.7. Statistical Analysis

Student *t*-test was used to assess the significance of the differences between values. A *P* value less than 0.05 was considered significant. The analysis of variance was used to evaluate the comparisons between groups using two-tailed analyses.

## 3. Results

### 3.1. Expression of Zinc Finger Proteins in Primary Articular Osteoarthritic Chondrocytes

**Figure 1:**
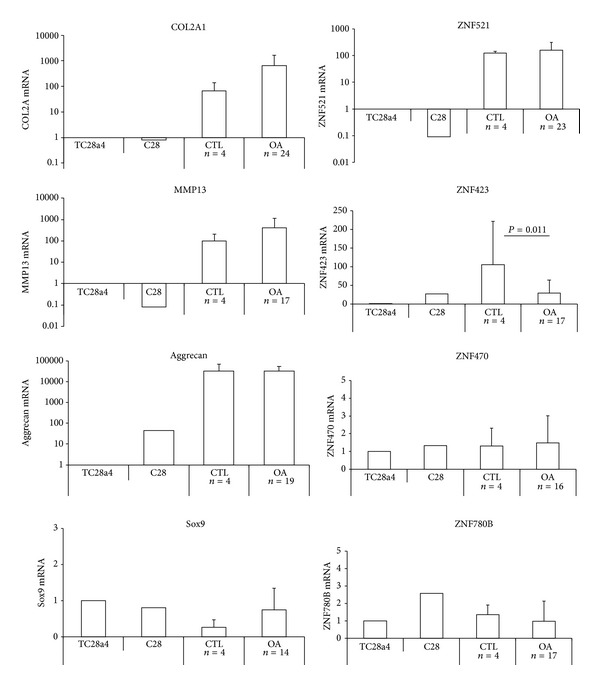
Expression of chondrocyte and zinc finger proteins in primary OA chondrocytes. Chondrocyte mRNA transcripts for COL2A, MMP13, aggrecan, and Sox9 were analysed with ZNF521, ZNF423, ZNF470, and ZNF780B in sets of control samples derived from hallux valgus and pes cavus and OA stages II–IV chondrocytes derived from patients undergoing total joint replacement. Numbers of patient specimens are indicated. mRNA levels were quantified by Q-RT-PCR normalized for GAPDH and calculated relative to the amount of each transcript in the chondrocyte cell line T/C28a4 (arbitrary units).

Chondrocytes from cartilage samples from either hip or knee joints of patients affected by OA (stages II–IV) undergoing replacement surgery were compared to a control group of samples from hallux valgus and pes cavus. The strategy used was to culture and amplify the chondrocytes for one passage to obtain sufficient RNA for a reliable Q-RT-PCR analysis. This strategy yielded results more reliably quantifiable than those obtained using RNA prepared directly from cartilage tissue, where the amount extracted was low and problems of degradation were more frequent. The relative amounts of mRNA present are compared to the chondrocyte-derived cell lines T/C-28a4 and the C-28 [33, 34] which are known to express lower levels of chondrocyte-specific genes. The transcripts encoding matrix proteins COL2A1 and aggrecan and the catalytic matrix-degrading protease MMP13 were expressed at considerably higher levels in the primary OA chondrocytes than in either chondrocyte cell line. In osteoarthritic cartilage quiescent chondrocytes become “activated” and proliferate, forming clusters and producing more of both matrix and matrix-degrading enzymes [1, 42]. This was confirmed by the expression of these genes in our group of specimens of OA chondrocytes (stages II–IV) that displayed higher expression levels of COL2A1 and MMP13 than control cells (Figure 1). Sox9, a transcription factor known to be required for chondrogenesis, was expressed at a similar level in the cell lines and in primary chondrocytes, indicating that it has not been lost when the cells were immortalized.

**Figure 2:**
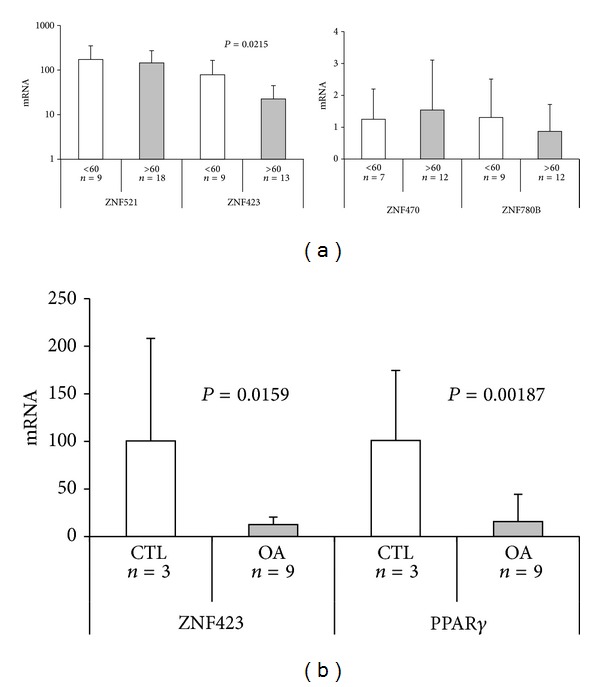
(a) Expression of zinc finger proteins in OA chondrocytes derived from patients according to age group. The OA patients were classified as under or over 60 years of age. Numbers of patient specimens are indicated. mRNA levels were quantified by Q-RT-PCR normalized for GAPDH and calculated relative to the amount of each transcript in the chondrocyte cell line T/C28a4 (arbitrary units). (b) Comparison of the transcripts for ZNF423 and PPAR*γ* in controls and OA chondrocytes. The expression of or ZNF423 and PPAR*γ* was compared in a set of 3 control (2 hallux valgus, 1 pes cavus) and 9 OA samples (stages III-IV) by Q-RT-PCR. The relative mRNA amounts are expressed as a percentage of the media of the control group.

Among the zinc finger proteins studied, ZNF521 was over 100-fold more expressed in primary chondrocytes, with no significant variation between control and OA cells, compared to the T/C-28a4 cell line (Figure 1). Such a high level of expression suggests that ZNF521 may be functionally relevant in primary chondrocytes. ZNF423 mRNA was also abundant in primary cells, but a significant decrease in the amounts of its transcript was observed in OA-derived chondrocytes compared to control cells (Figure 1; *P* = 0.011). In addition, the levels of ZNF423 mRNA showed a significant age-related variation, with a lower expression in chondrocytes derived from patients over 60 than under 60 years old (Figure 2(a); *P* = 0.0215). This trend was not evident for the other zinc finger proteins transcripts analyzed. ZNF470 and ZNF780B, like Sox9, were expressed at comparable levels in both OA and control primary chondrocytes as well as in the chondrocyte cell lines (Figure 1) and their expression did not change with age (Figure 2(a)).

The nuclear receptor, peroxisome proliferator-activated receptor gamma (PPAR*γ*), has been shown to exert an anti-inflammatory and chondroprotective action, and the modulation of its expression by interleukin 1*β*, or the cartilage-specific disruption of its gene in mice, has been linked to the development of OA [43, 44]. Since PPAR*γ* is a major target of ZNF423 in adipocyte precursors [27], we compared its expression in nine OA samples that showed low levels of ZNF423 mRNA to that of control samples and found a strong decrease in PPAR*γ* expression in OA chondrocytes, comparable to that of ZNF423 (Figure 2(b); *P* = 0.00187).

### 3.2. Expression of Zinc Finger Protein Genes during Dedifferentiation of Chondrocytes in Culture

Primary chondrocytes, once extracted from the cartilage matrix, can be cultured in adhesion on tissue culture plates. Initially two populations are visible: some cells are rounded and partly attached to the tissue culture dish and others adopt a more adherent form [3, 45]. In subsequent passages fewer round cells are present and the majority extend in a flattened fibroblastic fashion onto the dish. Over a period of 4-5 weeks with weekly passages the cell population becomes characteristically dedifferentiated. RNA was isolated at each passage during the cultures derived from 10 different OA chondrocyte samples and analyzed by Q-PCR for chondrocyte-specific and the zinc finger protein transcripts.

**Figure 3:**
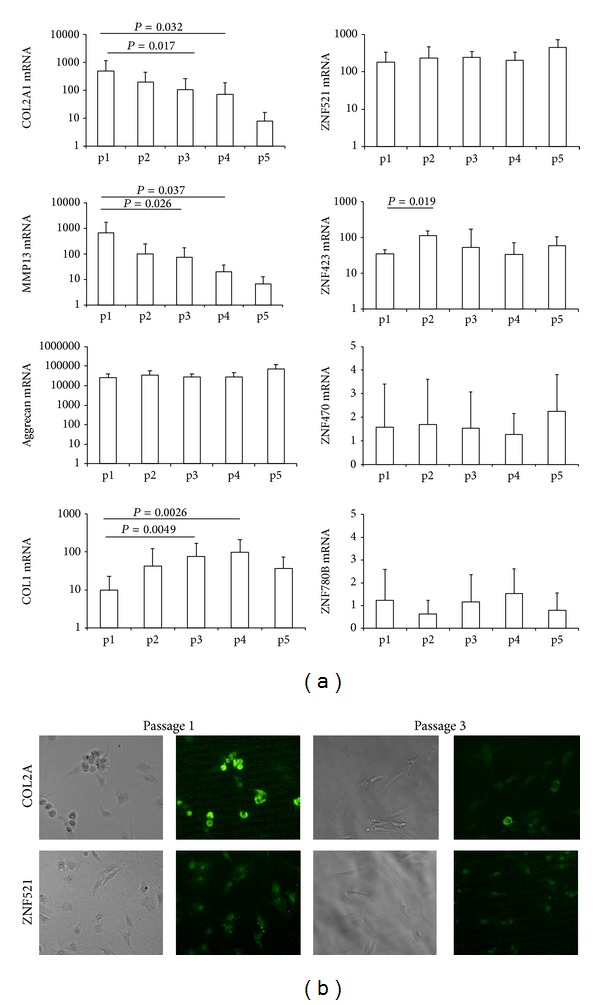
Expression of chondrocyte and zinc finger proteins during adherent chondrocyte passages. (a) Expression of the chondrocyte proteins COL2A1, MMP13, aggrecan, and COL1 as well as the zinc finger proteins ZNF521, ZNF423, ZNF470, and ZNF780B in adherent cultured chondrocytes. Each passage represents one week of growth on tissue culture treated dishes until the cells were 80% confluent. Cells were treated with trypsin and diluted at 1 : 3 for passage and RNA was prepared with Trizol. The expression of RNA levels was estimated by Q-RT-PCR normalized for GAPDH and calculated relative to the amount of each transcript in the chondrocyte cell line T/C28a4 (arbitrary units). Significant differences are indicated with *P* values less than 0.05. (b) Immunofluorescence of chondrocytes after one or three passages stained with rabbit antibodies specific for COL2A1 or ZNF521 and detected with anti-rabbit Alexa Fluor 495 (magnification 20x).

This analysis showed that the mRNAs for the chondrocyte matrix protein COL2A1 and the MMP13 protease decreased significantly during the cell culture passages, whereas aggrecan remained expressed at high levels throughout the culture period. Collagen type I, known to be associated with a more undifferentiated phenotype, notably increased as expected. We observed that the zinc finger proteins ZNF521 and ZNF423, which are expressed at very high levels compared to the chondrocytic cell line T/C28a4, continue to be expressed throughout the dedifferentiation culture period, whereas ZNF470 and ZNF780B retained expression levels comparable to those of T/C28a4 cells (Figure 3(a)).

Immunofluorescence staining for COL2A1 showed at early passages a high number of intensely staining cells in the rounded population, which was largely lost at later passages with most cells displaying only a considerably weaker staining. ZNF521 was expressed in the nucleus of both types of cells throughout the cultures (Figure 3(b)) confirming the results from the Q-PCR analysis.

### 3.3. Silencing of ZNF521 Prevents the Maintenance of a Differentiated Phenotype in Alginate Chondrocyte Cell Cultures

**Figure 4:**
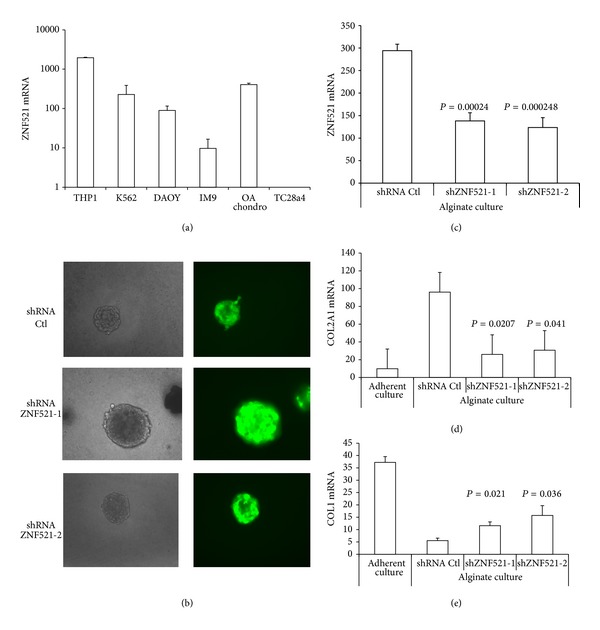
Silencing of ZNF521 in alginate chondrocyte cultures. (a) Comparison by Q-RT-PCR of the ZNF521 expression in OA chondrocytes (stage III), hematopoietic cell lines (THP1, K562, and IM9), a medulloblastoma cell line (DAOY), and the T/C28a4 chondrocyte cell line. (b) Alginate cultured cells were transduced with lentivirus expressing shRNA and the transgene EGFP; cells were directly visualized for green fluorescence at 20x magnification. (c) Downregulation of ZNF521 mRNA by shRNA lentiviral transduction of chondrocytes in alginate beads. After 3 weeks of culture in alginate beads Q-RT-PCR was performed on the RNA derived from either the control nontarget shRNA or the two shRNA specific for ZNF521. ((d) and (e)) Modulation of the COL2A1 and COL1 ratio by shRNA specific for ZNF521. Chondrocytes from stage III OA were cultured in adherence for 2 passages of trypsinization or in alginate beads with weekly medium changes. At 3 weeks cells were recovered by dissolving the matrix with EDTA and RNA was extracted for Q-RT-PCR and amplified with primers for COL2A1 and COL1.

The expression of ZNF521 in primary OA chondrocytes (Figure 4(a)) was considerably high and comparable to that observed in the hematopoietic cell lines THP1, K562 and in the medulloblastoma cell line DAOY, which have been shown to depend on a functionally active ZNF521 [7, 10, 46]. Therefore, an RNAi-based silencing approach was undertaken to assess the role of this protein in cultured primary chondrocytes. To this end, we infected primary chondrocytes with lentiviral vectors constructed to enable the integration in the target cells' genome of shRNAs specific for ZNF521 together with EGFP as a transgene, resulting in EGFP-identifiable cells that produce reduced amounts of ZNF521 mRNA (Figure 4(c)) and protein. Experiments were performed using chondrocytes encapsulated in alginate beads, a matrix in which chondrocytes grow in sphere-like bodies of associated cells, where EGFP-expressing cells could be visualised (Figure 4(b)). After 3 weeks of culture RNA was extracted and the expression of collagens was compared to that of cells grown in adhesion with weekly passages. The control chondrocytes in alginate beads had retained the differentiated phenotype compared to their counterparts growing in adherence, as shown by the significantly higher COL2A1 and lower COL1 expression. Instead, the cells transduced with either shRNA specific for ZNF521 displayed a more dedifferentiated phenotype, with a decrease in the COL2A1 mRNA and an increase in COL1 transcript, compared to control cells (Figures 4(d) and 4(e)).

## 4. Discussion

In OA the healthy balance of the cartilage homeostasis, normally ensured by articular chondrocytes present within the extra-cellular matrix, is disrupted. Chondrocytes embedded in the cartilage matrix normally produce the proteoglycans that compose the matrix, whose damage renders the cartilage tissue vulnerable to protease digestion. The changes in cellular activity such as those observed in the development of OA may be the consequence of discrete changes in the expression of sets of transcription factors that in turn control the expression of genes whose products are responsible for the maintenance of the physiological tissue homeostasis.

Proteins belonging to the Sox and Runx families are known to be central players in regulating transcription in chondrogenesis and differentiated chondrocytes present in the articulation cartilage. Sox9 is necessary for chondrogenic differentiation before and after mesenchymal condensations, whereas Sox5 and Sox6 are needed only after mesenchymal condensation [47]. Sox9 binds a consensus sequence in the collagen II enhancer region and activates expression. Haploinsufficiency of Sox9 in humans leads to the skeletal dysmorphology syndrome campomelic dysplasia [48] underlining the crucial role described for Sox9. Runx2 is known not only to be important in osteogenesis but also in articular cartilage and OA, where it induces the expression of MMP13 in a process that can be counteracted by TGFbeta [49–51], and its expression increases with the progression of OA resulting in a more pronounced matrix degradation by MMP13 [52]. Other factors including Shox/Shox2, Dlx5, and MEF2C have been also implicated particularly in the hypertrophy of chondrocytes and may have a role in pathogenesis of OA [53]. It is predicted that additional transcription factors may participate in the control of chondrocyte homeostasis and that an altered balance of their expression/activity is likely to contribute to its disruption that results in the onset of OA. We have focused our attention on a group of zinc finger proteins that, based on their established role in orchestrating the lineage fate choice of mesenchymal stem cells (ZNF423 and ZNF521) or on their direct or indirect links with cartilage physiology (ZNF470 and of ZNF780B), might play a regulatory role in normal articular chondrocytes and in OA.

The expression of these zinc finger proteins was investigated in chondrocytes from an extensive cohort of patients affected by OA at different stages, in comparison with chondrocytes derived from patients with disorders distinct from OA proper, considered as controls. The analyses were conducted both on freshly isolated cells (after one round of culture, necessary to achieve acceptable RNA purity) and on cells cultured for different periods of time in different conditions. While the expression profiles of ZNF470 and of ZNF780B did not display significant variations in any of the conditions analyzed, ZNF423 mRNA was significantly lower in those from OA compared to controls. This was paralleled by a similar strong decrease in the expression of the ZNF423 target, PPAR*γ*, a nuclear receptor whose inhibition has been associated with the development of OA [43, 44]. Whether this decrease is a direct consequence of the low expression of ZNF423 and whether enforced expression of ZNF423 in OA chondrocytes may restore normal levels of PPAR*γ* and attenuate the OA phenotype remain to be determined in future studies. It will also be of interest to conduct an in-depth analysis of the expression and activity of other factors, such as WISP2 and EBF1, which have been shown to functionally interact with ZNF423 in the control of PPAR*γ* expression [11, 27, 28].

In addition to OA, we found that ZNF423 was also significantly reduced in elderly patients (over 60) compared to younger ones. Cartilage chondrocytes are known to be extremely long lived and undergo changes with age, which result in modifications in their anabolic and catabolic processes and include a reduced responsiveness to growth factors, such as TGFbeta, BMPs, and WNT [54]. Since ZNF423 has been described to be a prominent effector of BMP2 and BMP4 [19, 55], a lower expression could be part of this mechanism. It must be kept in mind that all four controls, whose chondrocytes show higher levels of ZNF423 than the OA patients, are in the younger group; however, even if these subjects were excluded, the ZNF423 expression of the younger patients remained significantly higher than that of the elderly cohort. Taken together, the results illustrated in this paper suggest that ZNF423 underexpression may represent a useful OA-associated biomarker and possibly be a factor in OA pathogenesis. In this regard, it is interesting to notice that a recent in silico analysis of differentially coexpressed genes identified ZNF423 among the ten top-ranked transcription factors functionally relevant to OA [56].

The expression of ZNF521 did not display significant changes among the different patients' groups classified by age or OA stage; however, we were intrigued by the high levels of expression in primary chondrocytes compared to the immortalized cell lines. This prompted us to test whether silencing of ZNF521 expression would modify the phenotype of primary cells. As illustrated in Figure 3, ZNF521 knockdown with two distinct shRNAs in chondrocytes cultured in alginate beads resulted in a phenotype with marked characteristics of dedifferentiation. This suggests that the presence of high levels of ZNF521 expression may be a requisite for the maintenance of the identity in primary chondrocytes.

Mechanistically, transcription is controlled by the concerted action of transcription factors and epigenetic regulators, including high-mobility group proteins like HMGB-1 [57] and HMGA1 [35, 58] that are both increased in later stages of OA reflecting multiple changes at the level of transcription and an involvement in pathogenesis of OA, as well as histone-modifying enzymes such as histone acetylases (HATs) and deacetylases (HDACs). Several features of differentiated chondrocytes are known to be affected by the activity of these effectors; for instance, collagen type II expression is controlled by HDAC activity in articular chondrocytes [59] as well as by the class III HDAC, SirT1, a NAD-dependent HDAC, which regulates cartilage gene expression [60]. Both ZNF521 and ZNF423 have N-terminal motifs that interact with the HDAC-containing NuRD complex [5, 6, 61], and the integrity of this motif has been shown to be essential for the promotion of growth and tumorigenicity of medulloblastoma cells [10]; in addition, Zfp521 is also known to interact with HDAC3 and HDAC4 that are instrumental in the regulatory functions of Zfp521 in growth plate chondrocytes and osteoblasts [14, 15]. It will therefore be of interest to investigate whether the association of ZNF521 with chromatin-remodelling factors may also play a role in articular chondrocytes and in determining the OA phenotype. Finally, the functional interactions between ZNF521 and ZNF423 in articular chondrocytes warrant further investigation to determine whether the two factors exert antagonistic functions in these cells as documented in mesenchymal stem cells [11] or if their coexpression is required for the maintenance of the differentiated phenotype. A better understanding of the role of these factors in chondrocyte physiology may provide further insight into the molecular mechanisms that control their homeostasis and their potential contribution to the pathophysiology of OA.

## Figures and Tables

**Table 1:** Cartilage samples used in the study.

Patient	Age	BMI	Stage	Disease
1	47	26.18	Control	Hallux valgus
2	49	28.31	Control	Hallux valgus
3	58	32.81	Control	Hallux valgus
4	49	27.8	Control	Pes cavus
5	51	32.9	OA II	Hip osteoarthritis
6	70	24.69	OA II	Knee osteoarthritis
7	67	26.38	OA III	Hip osteoarthritis
8	63	27.8	OA III	Hip osteoarthritis
9	73	29.93	OA III	Knee osteoarthritis
10	65	28.02	OA III	Knee osteoarthritis
11	57	41.02	OA III	Knee osteoarthritis
12	71	27.4	OA III	Knee osteoarthritis
13	76	25.73	OA III	Knee osteoarthritis
14	73	28.37	OA III	Knee osteoarthritis
15	78	26.02	OA III	Knee osteoarthritis
16	74	24.22	OA III	Knee osteoarthritis
17	67	36.32	OA III	Knee osteoarthritis
18	66	29.33	OA III	Knee osteoarthritis
19	67	29.41	OA III	Knee osteoarthritis
20	67	29.41	OA III	Knee osteoarthritis
21	72	31.3	OA III	Knee osteoarthritis
22	72	31.3	OA III	Knee osteoarthritis
23	47	24.61	OA IV	Hip osteoarthritis
24	75	33.33	OA IV	Hip osteoarthritis
25	78	26.05	OA IV	Hip osteoarthritis
26	77	29.3	OA IV	Hip osteoarthritis
27	59	34.6	OA IV	Knee osteoarthritis
28	58	31.14	OA IV	Knee osteoarthritis
29	74	37.5	OA IV	Knee osteoarthritis

## References

[1] Goldring MB (2012). Chondrogenesis, chondrocyte differentiation, and articular cartilage metabolism in health and osteoarthritis. *Therapeutic Advances in Musculoskeletal Disease*.

[2] Harris JD, Siston RA, Pan X, Flanigan DC (2010). Autologous chondrocyte implantation: a systematic review. *Journal of Bone and Joint Surgery A*.

[3] Lin Z, Willers C, Xu J, Zheng M-H (2006). The chondrocyte: biology and clinical application. *Tissue Engineering*.

[4] Cavallo C, Desando G, Facchini A, Grigolo B (2010). Chondrocytes from patients with osteoarthritis express typical extracellular matrix molecules once grown onto a three-dimensional hyaluronan-based scaffold. *Journal of Biomedical Materials Research A*.

[5] Bond HM, Mesuraca M, Carbone E (2004). Early hematopoietic zinc finger protein (EHZF), the human homolog to mouse Evi3, is highly expressed in primitive human hematopoietic cells. *Blood*.

[6] Bond HM, Mesuraca M, Amodio N (2008). Early hematopoietic zinc finger protein-zinc finger protein 521: a candidate regulator of diverse immature cells. *International Journal of Biochemistry and Cell Biology*.

[7] Matsubara E, Sakai I, Yamanouchi J (2009). The role of zinc finger protein 521/early hematopoietic zinc finger protein in erythroid cell differentiation. *Journal of Biological Chemistry*.

[8] Mega T, Lupia M, Amodio N (2011). Zinc finger protein 521 antagonizes early B-cell factor 1 and modulates the B-lymphoid differentiation of primary hematopoietic progenitors. *Cell Cycle*.

[9] Kamiya D, Banno S, Sasai N (2011). Intrinsic transition of embryonic stem-cell differentiation into neural progenitors. *Nature*.

[10] Spina R, Filocamo G, Iaccino E (2013). Critical role of zinc finger protein 521 in the control of growth, clonogenicity and tumorigenic potential of medulloblastoma cells. *Oncotarget*.

[11] Kang S, Akerblad P, Kiviranta R (2012). Regulation of early adipose commitment by Zfp521. *PLOS Biology*.

[12] Kiviranta R, Yamana K, Saito H (2013). Coordinated transcriptional regulation of bone homeostasis by Ebf1 and Zfp521 in both mesenchymal and hematopoietic lineages. *The Journal of Experimental Medicine*.

[13] Wu M, Hesse E, Morvan F (2009). Zfp521 antagonizes Runx2, delays osteoblast differentiation in vitro, and promotes bone formation in vivo. *Bone*.

[14] Hesse E, Saito H, Kiviranta R (2010). Zfp521 controls bone mass by HDAC3-dependent attenuation of Runx2 activity. *Journal of Cell Biology*.

[15] Correa D, Hesse E, Seriwatanachai D (2010). Zfp521 is a target gene and key effector of parathyroid hormone-related peptide signaling in growth plate chondrocytes. *Developmental Cell*.

[16] Tsai RY, Reed RR (1997). Cloning and functional characterization of Roaz, a zinc finger protein that interacts with O/E-1 to regulate gene expression: implications for olfactory neuronal development. *The Journal of Neuroscience*.

[17] Tsai RYL, Reed RR (1998). Identification of DNA recognition sequences and protein interaction domains of the multiple-Zn-finger protein Roaz. *Molecular and Cellular Biology*.

[18] Cheng LE, Zhang J, Reed RR (2007). The transcription factor Zfp423/OAZ is required for cerebellar development and CNS midline patterning. *Developmental Biology*.

[19] Hata A, Seoane J, Lagna G, Montalvo E, Hemmati-Brivanlou A, Massagué J (2000). OAZ uses distinct DNA- and protein-binding zinc fingers in separate BMP- Smad and Olf signaling pathways. *Cell*.

[20] Huang S, Laoukili J, Epping MT (2009). ZNF423 is critically required for retinoic acid-induced differentiation and is a marker of neuroblastoma outcome. *Cancer Cell*.

[21] Masserdotti G, Badaloni A, Green YS (2010). ZFP423 coordinates Notch and bone morphogenetic protein signaling, selectively up-regulating Hes5 gene expression. *Journal of Biological Chemistry*.

[22] Warming S, Rachel RA, Jenkins NA, Copeland NG (2006). Zfp423 is required for normal cerebellar development. *Molecular and Cellular Biology*.

[23] Alcaraz WA, Gold DA, Raponi E, Gent PM, Concepcion D, Hamilton BA (2006). Zfp423 controls proliferation and differentiation of neural precursors in cerebellar vermis formation. *Proceedings of the National Academy of Sciences of the United States of America*.

[24] Cheng LE, Reed RR (2007). Zfp423/OAZ participates in a developmental switch during olfactory neurogenesis. *Neuron*.

[25] Miyazaki K, Yamasaki N, Oda H (2009). Enhanced expression of p210BCR/ABL and aberrant expression of Zfp423/ZNF423 induce blast crisis of chronic myelogenous leukemia. *Blood*.

[26] Harder L, Eschenburg G, Zech A (2013). Aberrant ZNF423 impedes B cell differentiation and is linked to adverse outcome of ETV6-RUNX1 negative B precursor acute lymphoblastic leukemia. *The Journal of Experimental Medicine*.

[27] Gupta RK, Arany Z, Seale P (2010). Transcriptional control of preadipocyte determination by Zfp423. *Nature*.

[28] Hammarsted A, Hedjazifar S, Jenndahl L (2013). WISP2 regulates preadipocyte commitment and PPAR*γ* activation by BMP4. *Proceedings of the National Academy of Sciences of the United States of America*.

[29] Perez M, Rompato G, Corbi N, De Gregorio L, Dragani TA, Passananti C (1996). Zfp60, a mouse zinc finger gene expressed transiently during in vitro muscle differentiation. *FEBS Letters*.

[30] Ganss B, Kobayashi H (2002). The zinc finger transcription factor Zfp60 is a negative regulator of cartilage differentiation. *Journal of Bone and Mineral Research*.

[31] Hering TM, Kazmi NH, Huynh TD (2004). Characterization and chondrocyte differentiation stage-specific expression of KRAB zinc-finger protein gene ZNF470. *Experimental Cell Research*.

[32] Lin L, Shen Q, Xue T, Duan X, Fu X, Yu C (2014). Sonic hedgehog improves redifferentiation of dedifferentiated chondrocytes for articular cartilage repair. *PLoS ONE*.

[33] Finger F, Schörle C, Zien A, Gebhard P, Goldring MB, Aigner T (2003). Molecular phenotyping of human chondrocyte cell lines T/C-28a2, T/C-28a4, and C-28/I2. *Arthritis and Rheumatism*.

[34] Goldring MB, Birkhead JR, Suen L-F (1994). Interleukin-1*β*-modulated gene expression in immortalized human chondrocytes. *Journal of Clinical Investigation*.

[35] Gasparini G, Gori MD, Paonessa F, Chiefari E, Brunetti A, Galasso O (2012). Functional relationship between high mobility group A1 (HMGA1) protein and insulin-like growth factor-binding protein 3 (IGFBP-3) in human chondrocytes. *Arthritis Research & Therapy*.

[36] Altman R, Alarcon G, Appelrouth D (1991). The American College of Rheumatology criteria for the classification and reporting of osteoarthritis of the hip. *Arthritis and Rheumatism*.

[37] Altman R, Asch E, Bloch D (1986). Development of criteria for the classification and reporting of osteoarthritis. Classification of osteoarthritis of the knee. *Arthritis and Rheumatism*.

[38] Ahlbäck S (1986). Osteoarthrosis of the knee. A radiographic investigation. *Acta Radiologica: Diagnosis*.

[39] Dehne T, Schenk R, Perka C (2010). Gene expression profiling of primary human articular chondrocytes in high-density micromasses reveals patterns of recovery, maintenance, re- and dedifferentiation. *Gene*.

[40] de Ceuninck F, Lesur C, Pastoureau P, Caliez A, Sabatini M (2004). Culture of chondrocytes in alginate beads. *Methods in Molecular Medicine*.

[41] la Rocca R, Fulciniti M, Lakshmikanth T (2009). Early hematopoietic zinc finger protein prevents tumor cell recognition by natural killer cells. *Journal of Immunology*.

[42] Goldring MB, Marcu KB (2009). Cartilage homeostasis in health and rheumatic diseases. *Arthritis Research and Therapy*.

[43] Afif H, Benderdour M, Mfuna-Endam L (2007). Peroxisome proliferator-activated receptor *γ*1 expression is diminished in human osteoarthritic cartilage and is downregulated by interleukin-1*β* in articular chondrocytes. *Arthritis Research and Therapy*.

[44] Vasheghani F, Monemdjou R, Fahmi H (2013). Adult cartilage-specific peroxisome proliferator-activated receptor gamma knockout mice exhibit the spontaneous osteoarthritis phenotype. *The American Journal of Pathology*.

[45] Thirion S, Berenbaum F (2004). Culture and phenotyping of chondrocytes in primary culture. *Methods in molecular medicine*.

[46] Fleischmann KK, Pagel P, Schmid I, Roscher AA (2014). RNAi-mediated silencing of MLL-AF9 reveals leukemia-associated downstream targets and processes. *Molecular Cancer*.

[47] Ikeda T, Kawaguchi H, Kamekura S (2005). Distinct roles of Sox5, Sox6, and Sox9 in different stages of chondrogenic differentiation. *Journal of Bone and Mineral Metabolism*.

[48] Bi W, Huang W, Whitworth DJ (2001). Haploinsufficiency of Sox9 results in defective cartilage primordia and premature skeletal mineralization. *Proceedings of the National Academy of Sciences of the United States of America*.

[49] Wang X, Manner PA, Horner A, Shum L, Tuan RS, Nuckolls GH (2004). Regulation of MMP-13 expression by RUNX2 and FGF2 in osteoarthritic cartilage. *Osteoarthritis and Cartilage*.

[50] Chen CG, Thuillier D, Chin EN, Alliston T (2012). Chondrocyte-intrinsic Smad3 represses Runx2-inducible matrix metalloproteinase 13 expression to maintain articular cartilage and prevent osteoarthritis. *Arthritis & Rheumatology*.

[51] Galasso O, Familiari F, de Gori M, Gasparini G (2012). Recent findings on the role of gelatinases (matrix metalloproteinase-2 and -9) in osteoarthritis. *Advances in Orthopedics*.

[52] Orfanidou T, Iliopoulos D, Malizos KN, Tsezou A (2009). Involvement of SOX-9 and FGF-23 in RUNX-2 regulation in osteoarthritic chondrocytes. *Journal of Cellular and Molecular Medicine*.

[53] Solomon LA, Bérubé NG, Beier F (2008). Transcriptional regulators of chondrocyte hypertrophy. *Birth Defects Research C: Embryo Today*.

[54] Li Y, Wei X, Zhou J J, Wei L (2013). The age-related changes in cartilage and osteoarthritis. *BioMed Research International*.

[55] Ku M, Howard S, Ni W, Lagna G, Hata A (2006). OAZ regulates bone morphogenetic protein signaling through Smad6 activation. *Journal of Biological Chemistry*.

[56] Li G, Han N, Li Z, Lu Q (2013). Identification of transcription regulatory relationships in rheumatoid arthritis and osteoarthritis. *Clinical Rheumatology*.

[57] Terada C, Yoshida A, Nasu Y (2011). Gene expression and localization of high-mobility group box chromosomal protein-1 (HMGB-1)in human osteoarthritic cartilage. *Acta Medica Okayama*.

[58] Galasso O, de Gori M, Nocera A, Brunetti A, Gasparini G (2011). Regulatory functions of insulin-like growth factor binding proteins in osteoarthritis. *International Journal of Immunopathology and Pharmacology*.

[59] Yun HH, Ryu J-H, Chun J-S (2007). Regulation of type II collagen expression by histone deacetylase in articular chondrocytes. *Journal of Biological Chemistry*.

[60] Dvir-Ginzberg M, Gagarina V, Lee E-J, Hall DJ (2008). Regulation of cartilage-specific gene expression in human chondrocytes by SirT1 and nicotinamide phosphoribosyltransferase. *Journal of Biological Chemistry*.

[61] Lin AC, Roche AE, Wilk J, Svensson EC (2004). The N termini of Friend of GATA (FOG) proteins define a novel transcriptional repression motif and a superfamily of transcriptional repressors. *Journal of Biological Chemistry*.

